# Ginsenoside Rg3 Inhibits the Growth of Osteosarcoma and Attenuates Metastasis through the Wnt/*β*-Catenin and EMT Signaling Pathway

**DOI:** 10.1155/2020/6065124

**Published:** 2020-07-11

**Authors:** Xiaohan Mao, Yaqian Jin, Tianyu Feng, Hao Wang, Dan Liu, Zhangxu Zhou, Qi Yan, Huini Yang, Jieru Yang, Jing Yang, Yan Ye, Yuxi Su, Guowei Zuo

**Affiliations:** ^1^Key Laboratory of Laboratory Medical Diagnostics of Ministry of Education, Chongqing Medical University, Chongqing 400016, China; ^2^Faculty of Basic Medical Sciences, Chongqing Medical University, Chongqing 400016, China; ^3^Department II of Orthopaedics, Chongqing Key Laboratory of Pediatrics, Ministry of Education Key Laboratory of Child Development and Disorders, National Clinical Research Center for Child Health and Disorders, China International Science and Technology Cooperation Base of Child Development and Critical Disorders, Children's Hospital of Chongqing Medical University, Chongqing, China

## Abstract

Osteosarcoma (OS) is the most common primary malignant bone cancer. An increasing number of studies have demonstrated that ginsenoside Rg3 (Rg3), which is extracted from the roots of the traditional Chinese herb Panax ginseng, plays a tumor suppression role in various malignant tumors. In the present study, we aimed at investigating the role of Rg3 in the proliferation, migration, and invasion of OS and at exploring the underlying mechanisms. Cell viability and proliferation were observed by MTT assay and crystal violet staining. The migration and invasion of cells were measured by wound-healing assay and Transwell method. Signaling pathway screening was investigated using luciferase reporter gene assay. qRT-PCR and western blot were performed to measure the expression of molecules involved in cell epithelial-mesenchymal transition (EMT), and Wnt/*β*-catenin pathway. Results suggested that Rg3 could not only inhibit proliferation but also hamper the migration and invasion of OS. qRT-PCR and western blot demonstrated that a reduced level of MMP2/MMP7/MMP9 was induced after Rg3 treatment. In addition, the expression levels of proteins related to EMT and the Wnt/*β*-catenin pathway were downregulated. In summary, our data revealed that Rg3 could inhibit the proliferation, migration, and invasion of OS cells. This effect of Rg3 might be mediated by downregulating MMP2, MMP7, and MMP9 expression and suppressing EMT as well as the Wnt/*β*-catenin pathway. Thus, Rg3 might be a potential agent for the treatment of OS.

## 1. Introduction

Osteosarcoma (OS), the most common type of bone cancer, is the second leading cause of cancer-related death in children and adolescents [1]. It represents an invasive malignancy, in which OS cells spread to other organs when uncontrolled [2]. Given the effects of radical surgery and chemotherapy, a remarkable reduction in the quality of life is found in children and adolescents [3]. Despite intensive chemotherapy, the survival rate of patients with OS has not changed over the past 30 years, mainly due to metastasis and recurrence [4]. However, traditional chemotherapy drugs have severe side effects and can cause resistance. To improve patient survival rate, an effective new drug for OS should be developed.

Ginsenoside Rg3, which is extracted from a traditional medical herb of ginseng, is a relatively safe drug with anticancer activity both in vitro and in vivo [5, 6]. Recently, more and more studies have demonstrated that Rg3 is involved in chemotherapy resistance in many cancers, making it a promising Chinese herbal monomer for oncotherapy. Rg3 has been reported to inhibit prostate cancer cell proliferation and induce cell cycle arrest [7]; reduce melanoma cell growth, metastasis, and melanoma-induced angiogenesis in vitro and in vivo through suppression of the ERK and Akt pathways [8]; and promote tumor cell apoptosis [9] and immunity [10]. In 2002, Rg3 was approved by the Chinese Food and Drug Administration for treating non-small-cell lung cancer [11]. Besides, Sun et al. and Lu et al. reported that combined chemotherapy with Rg3 can improve the survival of cancer patients with advanced non-small-cell lung cancer after surgery [12, 13]. When it is combined with paclitaxel, it enhances cytotoxicity and apoptosis through NF-*κ*B inhibition in human triple-negative breast cancer MDA-MB-231, MDA-MB-453, and BT-549 cells [14]. In the treatment of OS, chemotherapy resistance is one of the main causes of low cure rates for patients. These studies substantiate that Rg3 may play a vital role in tumor cell resistance. Li et al. found that Rg3 can inhibit the proliferation and migration of osteosarcoma (MG-63, U-2OS, and SaoS-2) and induce cell apoptosis [15]. In Li's previous research, they found that Rg3 can regulate the survival of pancreatic cancer cells through the phosphatidylinositol 3-kinase/Akt/mammalian rapamycin target (PI3K/Akt/mTOR) pathway [16]. Therefore, they hypothesized that the PI3K/Akt/mTOR pathway may be a potential mechanism for the invasive effect of Rg3 on OS. However, our study focuses on the role and mechanism of Rg3 on OS metastasis. So two malignant cell lines 143B and MG63 were selected. For the research of molecular mechanism, we used the luciferase reporter gene experiment to screen the signaling pathways, which is more reliable and convincing.

Therefore, the present study was performed to investigate the effect of Rg3 on the proliferation and metastasis of OS and the potential molecular mechanism.

## 2. Materials and Methods

### 2.1. Cell Culture and Reagents

OS cell lines MG63 and 143B were obtained from the American Type Culture Collection (Manassas, USA). Cells were cultured in DMEM (HyClone, New Zealand) containing 10% fetal bovine serum (FBS, Gibco Life Technologies, Carlsbad, USA) and a mixture of 1% streptomycin (100 U/mL) and penicillin (100 *μ*g/mL) (HyClone, New Zealand). The incubator with a humidified atmosphere of 5% CO_2_ and 95% air was used for cell culture. Ginsenoside Rg3 (purity > 98%) was purchased from Must Bio-Technology (Chengdu, China); the Rg3 powder was dissolved in DMSO and stored at −20°C.

### 2.2. Crystal Violet Staining

Cells were seeded in a 24-well plate at a density of 2 × 10^4^ cells. Subsequently, these plates were cultured in an incubator at 37°C under 5% CO_2_. The next morning, cells were treated with different concentrations of Rg3 for 24, 48, and 72 h. Cells were stained with crystal violet to visualize the cell viability. For quantification, 24-well plates were stained with crystal violet, and 200 *µ*L of 10% acetic acid was added to each well. The optical density of different groups was detected by using an enzyme immunoassay analyzer (Bio-Rad, Hercules, USA) at 595 nm. Cell viability was calculated using the following formula: cell viability (%) = experimental group absorbance value/control group absorbance value × 100%.

### 2.3. MTT Assay

Cell viability was measured by MTT assay. A total of 3 × 10^3^ cell solution was seeded in each well of the 96-well plates and then placed in an incubator for overnight culture. The next morning, cells were treated with different concentrations of Rg3 for 24, 48, and 72 h. Subsequently, 10 *μ*L of MTT (Solarbio, Beijing, China) solution was added into the well of the 96-well plates, and the cells were cultured for 4 h at 37°C. The optical density of different groups was detected by using an enzyme immunoassay analyzer (Bio-Rad, Hercules, USA) at 492 nm. Cell viability was calculated using the following formula: cell viability (%) = experimental group absorbance value/control group absorbance value × 100%.

### 2.4. Wound-Healing Assay

Cells were seeded in a 6-well plate at a density of 2 × 10^4^ cells. Subsequently, the plates were cultured in an incubator at 37°C under 5% CO_2_. When the cell confluence reached 100%, a cross was uniformly drawn at the bottom of each well using a 10 *μ*L tip. Next, the wells were washed twice with PBS to remove the exfoliated cells, and 1.5 mL of new DMEM containing 2% FBS was added. Finally, the cells were treated with different concentrations of Rg3 and photographed at 0, 12, and 24 h.

### 2.5. Migration and Invasion Assay

Filters (Corning, NY, USA) with a pore size of 8 *μ*m diameters were used to measure the migration and invasion ability of cells. Cells were collected and resuspended with a serum-free medium. The density of cells was adjusted to 1.25 × 10^5^/mL. Next, 200 *μ*L cell suspension, treated with different concentrations of Rg3, was added into the upper chamber, and 600 *μ*L of medium containing 10% FBS was added to the lower chamber. Cell culture was continued for 24 h. After that, the medium was aspirated and fixed with 4% paraformaldehyde for 20 min. Subsequently, the chambers were naturally dried and stained with 0.1% crystal violet for 15 min and then washed with PBS three times. For invasion ability measurement, the Matrigel was diluted by a serum-free medium at a rate of 1 : 16. Then, 80 *μ*L of diluted Matrigel was added into the membrane of the chamber and was placed into an incubator at 37°C for 1 h. After that, cells were seeded in chambers at a density of 2.5 × 10^4^ cells/well and treated with different concentrations of Rg3. Then, 600 *μ*L of medium containing 20% FBS was added to the lower chamber. Cell culture was continued for 24 h. Afterwards, the process of crystal violet staining is the same as before. Cells were counted under a microscope at ×200 magnification.

### 2.6. qRT-PCR

Total RNA was extracted from cells at 90% confluence by TRIzol (Invitrogen Life Technologies, Carlsbad, USA). Total RNA concentrations were measured using a NanoDrop 1000 Spectrophotometer (Thermo Scientific, USA). According to the protocol, 2 *μ*g of total RNA in each sample was reverse-transcribed by using the Reverse Transcriptase M-MLV (RNase H) Kit (Takara Biotechnology, Otsu, Japan). The cDNA after reverse transcription was diluted 5 times with ddH_2_O and stored at −20°C. qRT-PCR was performed using a SYBR Green RT-PCR Kit (Takara Biotechnology, Otsu, Japan), and specific primers were run in the CFX Connect™ Real-Time System. The relative expression of target genes was normalized to those of GAPDH and expressed as 2^−ΔΔCt^. The sequences of primers for qRT-PCR are listed in Table 1.

### 2.7. Transfection and Luciferase Reporter Assay

A series of cancer-related signaling pathway luciferase reporters was provided by Prof. Tong-Chuan He from the University of Chicago. Cells were seeded in 25 cm^2^ flasks. After the cell confluence reached 50%–60%, different luciferase reporters were transfected into these flasks by using the H4000 transfection reagent (Engreen, Beijing, China). After 24 h, cells were collected and reseeded in 24-well plates at a density of 4 × 10^4^/well. The next morning, altered concentrations of Rg3 were added into each group of the 24-well plates for 24 h. Each group had three wells. Afterwards, 10 *μ*L of culture medium supernatant was taken for Gaussia luciferase assay utilizing the BioLux Gaussia Luciferase Assay Kit (NEB, USA).

### 2.8. Western Blot Assay

Cells treated with Rg3 for 48 h were collected and lysed with RIPA buffer (Beyotime Biotechnology, Shanghai, China), containing protease inhibitors and phosphatase inhibitors (Roche Diagnostics, Indianapolis, IN, USA). The concentration of each sample was calculated by using the BCA Protein Assay Kit (Beyotime Biotechnology, Shanghai, China). The protein solution was mixed with 5× SDS buffer and boiled in boiling water for 5 to 10 min. Approximately 30 *μ*g of total protein in each sample was loaded in the lane, separated by SDS-PAGE, and transferred to the polyvinylidene fluoride membrane (Millipore Corporation, Billerica, USA). The membranes were blocked by 5% skimmed milk, which was dissolved in 1× TBST wash buffer, at room temperature for 2 h. Subsequently, these membranes were washed with 1× TBST three times, each time for 10 min, and incubated overnight with the corresponding primary antibody at 4°C according to the best dilution rate (1 : 500∼1 : 1000). The next morning, the membranes were washed with TBST and incubated with secondary antibodies. The antibodies are following: rabbit anti-human MMP2, MMP7, and MMP9 (Affinity Biosciences, Zhenjiang, Jiangsu, China); rabbit anti-human E-cadherin and zinc finger E-box binding homeobox 1 (ZEB1) (ImmunoWay Biotechnology Company, Jiangsu, China); rabbit anti-human N-cadherin (Cell Signaling Technology, Danvers, MA, USA); rabbit anti-human Vimentin, Snail, Twist, *β*-catenin, c-Myc, and Cyclin D1 (Wanleibio, Shenyang, China); and mouse anti-human *β*-actin monoclonal antibody and secondary antibodies (Zhongshan Golden Bridge Biotechnology, Beijing, China). Immunoblots were carried out using enhanced chemiluminescence detection substrate (Billerica, MA, USA). The protein expression levels were normalized to *β*-actin as an internal control.

### 2.9. Statistical Analysis

The experimental data were analyzed by using SPSS 17.1 software and GraphPad Prism 5.0 software. All experiments were repeated three times and shown as mean ± standard deviations (SD). Statistical analyses were performed using one-way ANOVA with Dunn's multiple comparisons test. *P* < 0.05 was considered statistically significant.

## 3. Results

### 3.1. Rg3 Inhibits the Proliferation of OS Cells

**Figure 1:**
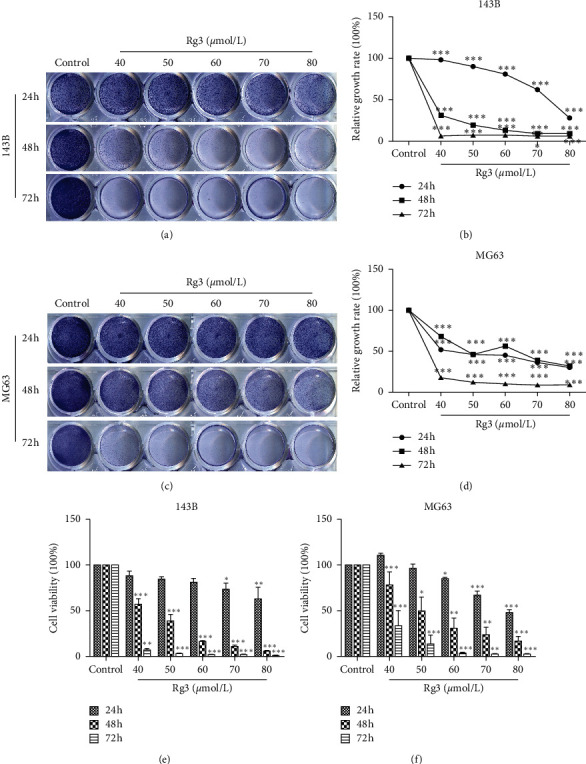
Effect of Rg3 on the proliferation ability of 143B and MG63 cells. The cells were treated with varying concentrations of Rg3 or DMSO for 24, 48, and 72 h. (a, c) Cell proliferation was observed by crystal violet staining. (b, d) Quantitative assessment of colony-forming rate at the indicated time (24, 48, and 72 h). (e, f) Cell proliferation of 143B and MG63 was measured by MTT assay. Data are shown as mean ± SD (*n* = 3). ^*∗*^*P* < 0.05 vs. control, ^*∗∗*^*P* < 0.01 vs. control, and ^*∗∗∗*^*P* < 0.001 vs. control.

The effect of Rg3 on the proliferation of OS cells was detected by crystal violet staining and MTT assay. As shown in Figures 1(a) and 1(c), compared with the control group, Rg3 could inhibit the proliferation of OS cells in dose- and time-dependent manners. The difference is statistically significant, as shown in Figures 1(b) and 1(d). A similar result was also obtained by MTT assay, as shown in Figures 1(e) and 1(f). According to the results of the MTT assay, the IC_50_ values in 143B and MG63 cells were 51 ± 3.46 and 57.07 ± 9.49 *μ*mol/L, respectively. Therefore, we selected three concentration treatment groups (143B: 40 *μ*mol/L for the low concentration group, IC_50_ group, and 60 *μ*mol/L for the high concentration group; MG63: 50 *μ*mol/L for the low concentration group, IC_50_ group, and 70 *μ*mol/L for the high concentration group) for the following research.

### 3.2. Rg3 Inhibits the Migration of OS Cells

**Figure 2:**
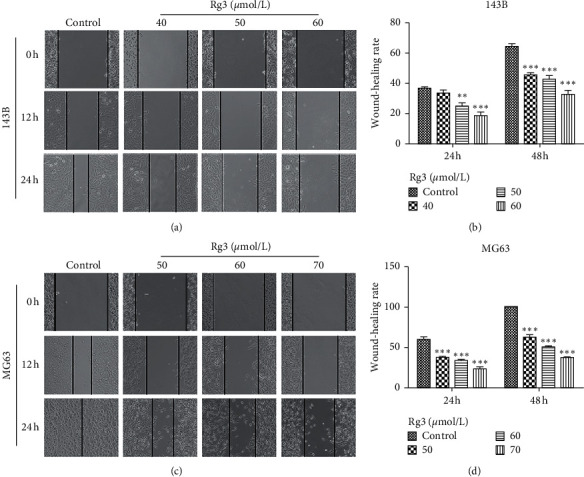
Effect of Rg3 on the migration ability of 143B and MG63 cells identified by wound-healing assay. These cells were treated with the indicated concentrations of Rg3 for 24 h. (a, c) 143B and MG63 cells were scraped and photographed at the indicated time (×100; 0, 12, and 24 h). (b, d) Wound-healing rate at the indicated times (12 and 24 h). Data are shown as mean ± SD (*n* = 3). ^*∗∗*^*P* < 0.01 vs. control and ^*∗∗∗*^*P* < 0.001 vs. control.

**Figure 3:**
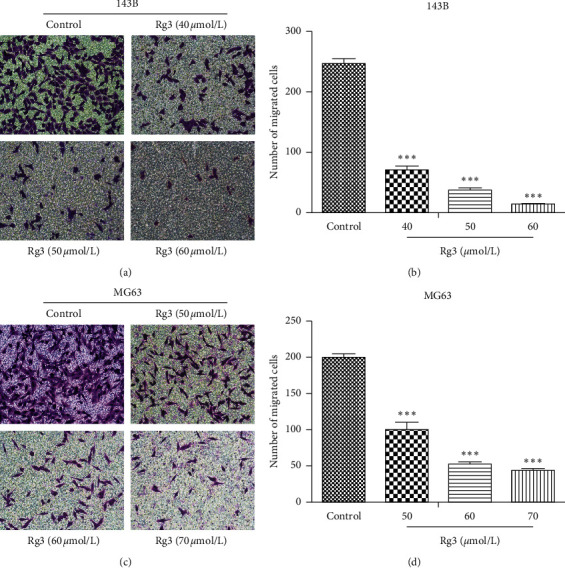
Effect of Rg3 on the migration ability of 143B and MG63 cells measured by the Transwell method. These cells were treated with Rg3 for 24 h. (a, c) The migration cells in different groups. (b, d) The number of migrated cells in each group. Data are shown as mean ± SD (*n* = 3). ^*∗∗∗*^*P* < 0.001 vs. control.

Wound-healing assay and Transwell assay were utilized to examine the effect of migration with Rg3 treatment. As shown in Figures 2(a) and 2(c), compared with the untreated group, the migration ability of OS cells was hampered by Rg3, and this negative effect was positively correlated with Rg3 concentration. The difference is statistically significant, as shown in Figures 2(b) and 2(d). When 143B and MG63 cells were treated with Rg3 (60 or 70 *μ*mol/L) for 24 h, the migration rate decreased to 32.61% and 37.13%, respectively. Moreover, the Transwell migration of OS cells was also blocked by Rg3. As shown in Figures 3(a) and 3(c), when Rg3 was added into the upper chamber of 24-well plates, the number of cells that migrated into the lower chamber decreased. These results suggested that Rg3 inhibited the migration of 143B and MG63 cells.

### 3.3. Rg3 Inhibits the Invasion Activity of OS Cells

**Figure 4:**
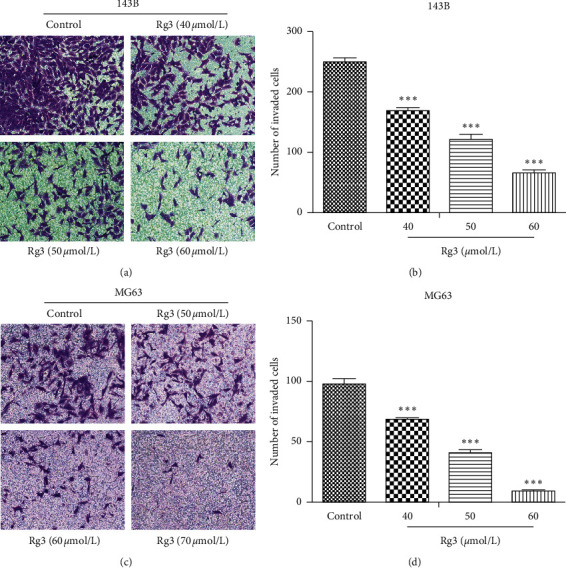
Effect of Rg3 on the invasion ability of 143B and MG63 cells discovered by Transwell. These cells were treated with Rg3 for 24 h. (a, c) The invasion cells in different groups. (b, d) The number of invasion cells in each group. Data are shown as mean ± SD (*n* = 3). ^*∗∗∗*^*P* < 0.001 vs. control.

To detect the invasion activity of 143B and MG63, Matrigel was precoated into the bottom membrane of the upper chamber. Compared with the control group, the invasion activity of OS cells was hampered when cells were treated with Rg3. As shown in Figure 4, the number of cells obtained through the Matrigel was remarkably decreased upon Rg3 treatment. These data indicated that Rg3 could attenuate the invasion activity of OS cells.

### 3.4. Rg3 Reduces the Expression of MMPs in OS Cells

**Figure 5:**
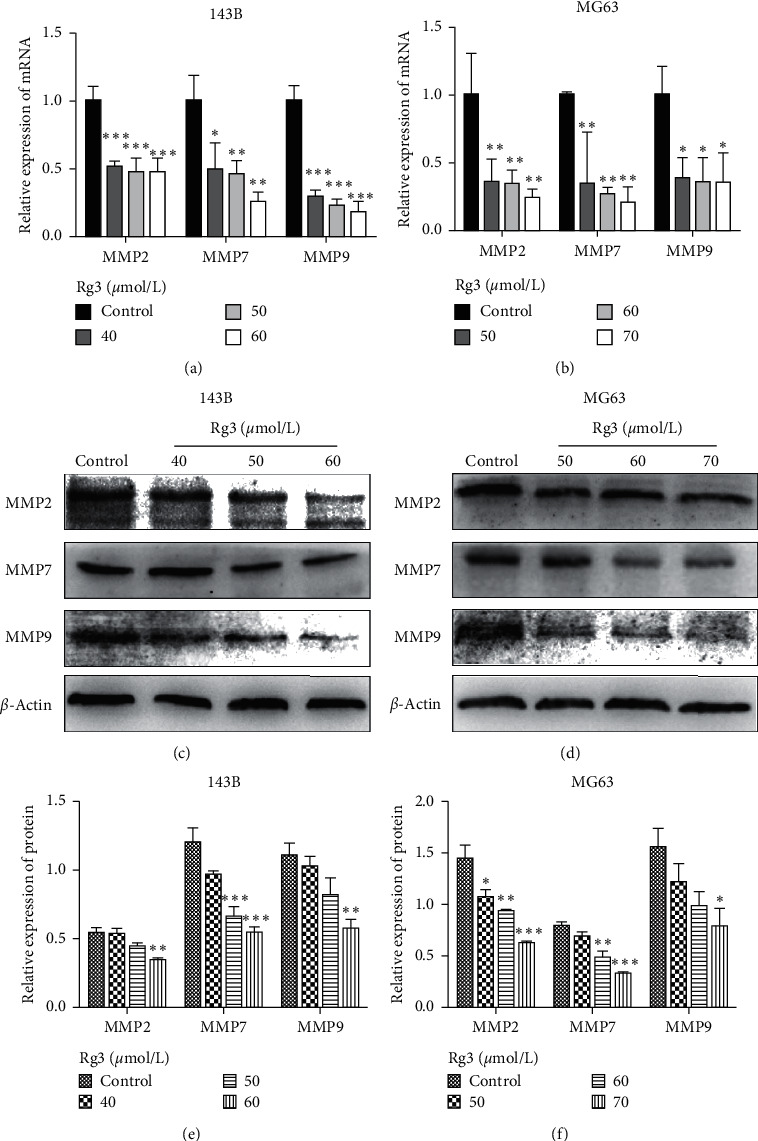
Effect of Rg3 on the expression levels of MMP2, MMP7, and MMP9 in human OS cells. (a, b) The mRNA expression of MMP2, MMP7, and MMP9 in 143B and MG63 cell lines. (c, d) The protein level of MMP2, MMP7, and MMP9 in 143B and MG63 shown by western blot. (e, f) Relative protein expression of MMP2, MMP7, and MMP9 in OS cells. Data are shown as mean ± SD (*n* = 3). ^*∗*^*P* < 0.05 vs. control, ^*∗∗*^*P* < 0.01 vs. control, and ^*∗∗∗*^*P* < 0.001 vs. control.

A crucial step for tumor cell extravasation and metastasis is the migration through the extracellular matrix, which requires proteolytic activity [17]. MMP2 and MMP9, which selectively degrade the major component of ECM, play a key role in the metastatic process [18]. MMP7 also takes part in metastasis. To explore the possible regulation effect of Rg3 on the expression of MMP2/MMP7/MMP9 in OS cells, we measured the mRNA and protein levels of MMP2/MMP7/MMP9 with Rg3 treatment. As shown in Figure 5, the mRNA and protein expression levels of MMP2/MMP7/MMP9 decreased. This finding suggested that Rg3 could inhibit the invasion of OS cells by downregulating the expression of MMP2, MMP7, and MMP9.

### 3.5. Rg3 Suppresses the Expression of Epithelial-Mesenchymal Transition (EMT) Markers and Associated Translation Factors

**Figure 6:**
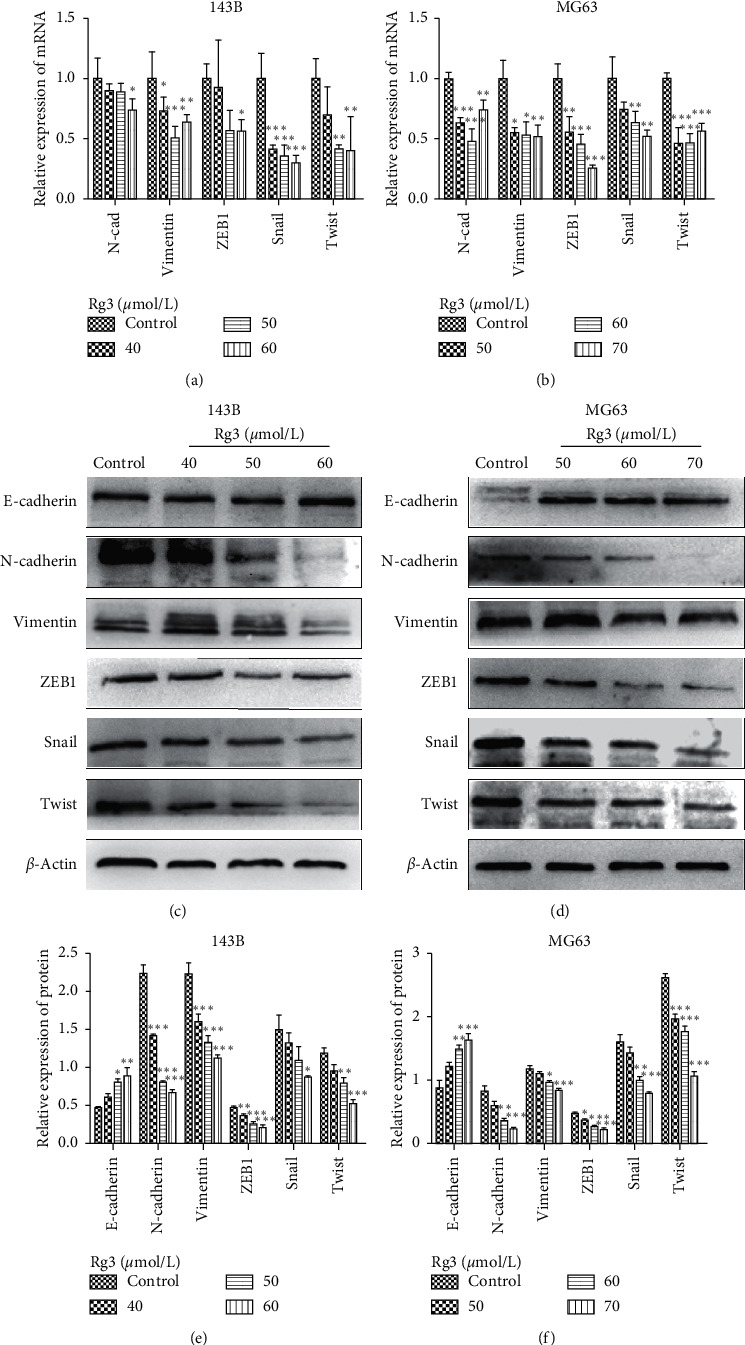
Effect of Rg3 on the expression levels of EMT-associated factors in human OS cells. (a, b) The mRNA expression of EMT in 143B and MG63 cell lines. (c, d) The protein levels of EMT in 143B and MG63. (e, f) Relative protein expression of EMT-related factors in OS cells. Data are shown as mean ± SD (*n* = 3). ^*∗*^*P* < 0.05 vs. control, ^*∗∗*^*P* < 0.01 vs. control, and ^*∗∗∗*^*P* < 0.001 vs. control.

An increasing number of studies have demonstrated that EMT has a vital relationship with cancer metastasis. To assess the influence of Rg3 on EMT makers, qRT-PCR and western blot were applied to measure the changes of EMT-related molecules upon Rg3 treatment. As shown in Figure 6, compared with the control group, mesenchymal markers, N-cadherin and Vimentin, decreased with Rg3 treatment. Given that EMT is driven by specific EMT-related transcription factors (EMT-TFs) [19], we determined whether EMT-TF expression is regulated by Rg3. As shown in Figures 6(a) and 6(b), the mRNA expression of Snail, Twist, and ZEB1 was inhibited by Rg3. This inhibitory effect of Rg3 was confirmed by western blot (Figures 6(c) and 6(d)). The aforementioned data supported the idea that Rg3 suppresses the EMT of OS cells, which may be mediated by inhibiting EMT-TFs.

### 3.6. Rg3 Inhibits the Activation of the Wnt/*β*-Catenin Signaling Pathway in OS Cells

**Figure 7:**
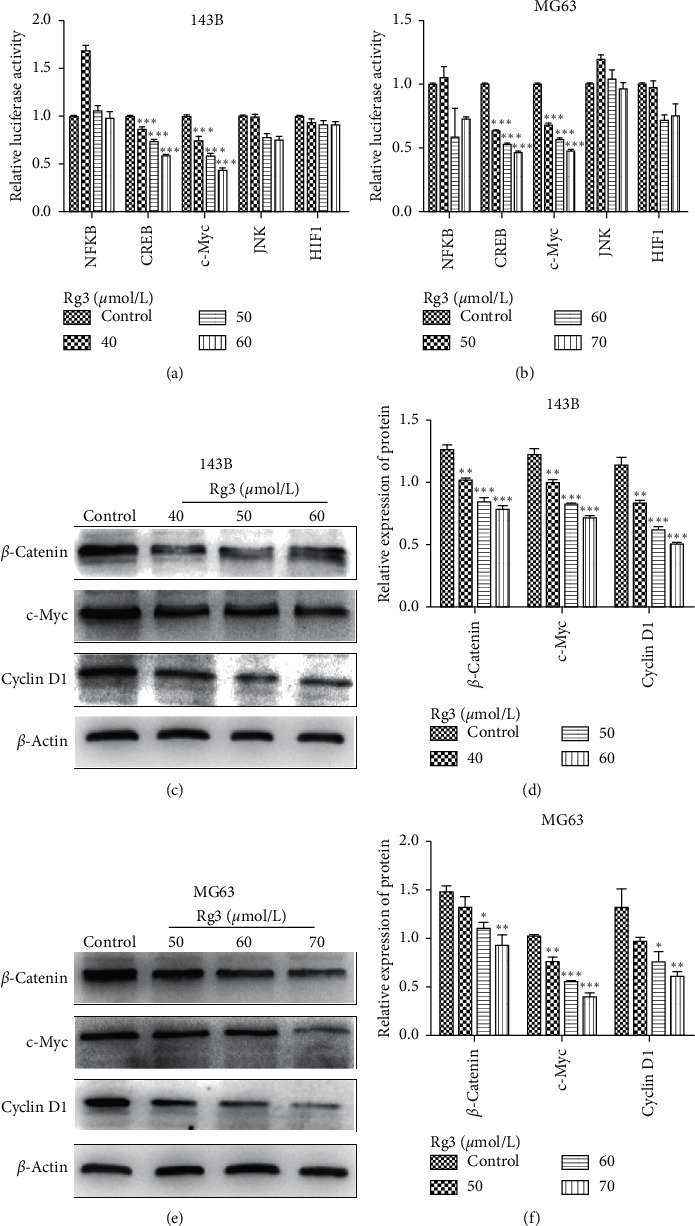
Effect of Rg3 on the Wnt/*β*-catenin signaling pathway. (a, b) The effects of Rg3 on the activity of different signaling pathways in 143B and MG63 cells were identified by luciferase reporter assay. (c, e) Western blot showing that Rg3 downregulated *β*-catenin, c-Myc, and Cyclin D1. (d, f) Relative protein expression of Wnt/*β*-catenin signaling pathway in OS cells. Data are shown as mean ± SD (*n* = 3). ^*∗*^*P* < 0.05 vs. control, ^*∗∗*^*P* < 0.01 vs. control, and ^*∗∗∗*^*P* < 0.001 vs. control.

Built on the above research, we aimed to have a better understanding of the aforementioned effects. Luciferase reporter gene assay was carried out to solve this problem. As shown in Figures 7(a) and 7(b), a downregulation of c-Myc-Luc, which contains c-Myc-responsive elements and represents c-Myc transcriptional activity, was observed. This result suggested that Rg3 may inhibit the Wnt/*β*-catenin signaling pathway. To verify this hypothesis, western blot was applied to assess the changes of the key factors of the Wnt/*β*-catenin signaling pathway (*β*-catenin, c-Myc, and Cyclin D1) at protein levels in OS cells. The opposite results were found in cells upon Rg3 treatment (Figures 7(c) and 7(e)). The data demonstrated that Rg3 negatively regulated the expression of *β*-catenin, c-Myc (downstream of *β*-catenin), and Cyclin D1. Our results revealed with some degree of certainty that Rg3 could obstruct OS cells by negatively regulating the Wnt/*β*-catenin signaling pathway.

## 4. Discussion

OS is a common malignant bone tumor that is a serious threat to the health of adolescents or children [20]. Despite the current standard therapy, surgical resection combined with postoperative chemotherapy, the survival rate still remains unsatisfactory. Drugs that are used in OS chemotherapy generally cause side effects and injuries in normal tissues of cancer patients [21]. In addition, metastasis is the common cause of death in OS. Therefore, finding a potential treatment drug to prevent and inhibit metastasis in OS patients is necessary.

Traditional Chinese medicines have been demonstrated to provide a rich resource for the identification of anticancer drugs [22]. Ginsenoside Rg3, a vital ingredient of the traditional Chinese herb Panax ginseng, has been declared to exert multiple anticancer effects in different cancers. At present, ginsenoside Rg3 is primarily used as a tumor angiogenesis inhibitor to prevent the recurrence and metastasis of various malignant tumors after traditional therapy due to its efficacy on the proliferation of tumor vascular endothelial cells and the formation of new blood vessels by regulating certain cytokine antiangiogenic factors [23]. However, few studies have concentrated on the anticancer effects of Rg3 in OS cells. Zhang et al. found that Rg3 induces DNA damage in human osteosarcoma cells and can protect normal human fibroblasts against the DNA damage and apoptosis induced by N-methyl-N′-nitro-N-nitrosoguanidine (MNNG) treatment *in vitro* [24]. Theoretically, ginsenosides would destroy cancer cells but leave normal cells unharmed. Thus, we evaluated the anticancer effects of Rg3 on 143B and MG63 cell lines. We first found that Rg3 could inhibit the proliferation of OS cells. In subsequent experiments, the results have shown that Rg3 has exceptional ability of inhibiting the migration and invasion of OS cells in a dose-dependent manner. Take into account these results, we hypothesized that Rg3 may inhibit the proliferation, migration, and invasion of OS cells.

Metastasis of malignant tumors is a primary problem that has to be overcome for tumor treatment. We found that Rg3 downregulated the expression of MMP2, MMP7, and MMP9 at mRNA and protein levels. Studies show that the depth of invasion, metastasis distance, and vascular permeability is positively correlated with the expression levels of MMP2, MMP7, and MMP9 [25]. Rg3 can suppress the expression of MMPs. Moreover, Rg3-induced downregulation of MMP9 is associated with the decreased invasive capacity of ovarian cancer cells [26]. Rg3 effectively inhibits the formation of pancreatic cancer vasculogenic mimicry by downregulating the expression of VE-cadherin, EphA2, MMP9, and MMP2 [27]. Our results are consistent with those of previous studies.

In addition, EMT in cancer is associated with tumor migration and invasion, stemness, survival, and therapy resistance. Classically, EMT has been thought to be critical for local invasion and cancer cell dissemination in the body [28]. During this process, cells lose their epithelial characteristics and acquire mesenchymal cell properties and subsequently show high metastatic potential [29]. In the present study, we investigated the changes of EMT-related proteins in Rg3-treated and untreated OS cells. We discovered that the expression of the epithelial marker E-cadherin was increased, whereas the expressions of mesenchymal markers, N-cadherin and Vimentin, were decreased. In addition, the function of EMT-TFs was blocked by Rg3. Therefore, Rg3 inhibited tumorigenesis by regulating EMT signals.

Moreover, many signaling pathways participate in EMT, such as TGF-*β*, Wnt/*β*-catenin, Ras, Notch, PI3K/AKT, and ERK pathways [30]. Aberrant activation or inactivation of the EMT-related signaling pathways is associated with tumorigenesis and metastasis [31]. In the current study, we found that Rg3 reduced the fluorescent activity of c-Myc luciferase plasmid (Figures 7(a) and 7(b)). Furthermore, Rg3 may regulate OS cells through the Wnt/*β*-catenin signaling pathway. This hypothesis was further confirmed by western blot (Figures 7(c) and 7(e)). Likewise, the major function of Cyclin D1 is to promote proliferation. Cyclin D1 is well known as a protooncogene, whose overexpression can make cells lose the control of proliferation, resulting in malignancy. Thus, our findings support the conclusion that Rg3 inhibits the proliferation and EMT of OS cells through the Wnt/*β*-catenin signaling pathway.

In summary, Rg3 can suppress the proliferation of OS cells and inhibit their migration and invasion abilities. These effects are exerted by decreasing the expression of MMPs, hampering EMT-related markers, and regulating the activity of the Wnt/*β*-catenin signaling pathway.

## 5. Conclusions

Our study demonstrated evidence that Rg3 could inhibit the proliferation, migration, and invasion of OS cells. Thus, Rg3 may be a potential anticancer agent for OS.

## Figures and Tables

**Table 1:** Primer sequences used for qRT-PCR.

Gene	Sequences (5′ ⟶ 3′)	
*GAPDH*	Forward	CAG CGA CAC CCA CTC CTC
	Reverse	TGA GGT CCA CCA CCC TGT

*MMP2*	Forward	AGA CAT ACA TCT TTG CTG GAG ACA
	Reverse	CTT GAA GAA GTA GCT GTG ACC G

*MMP7*	Forward	GGA GGA GAT GCT CAC TTC GAT
	Reverse	AGG AAT GTC CCA TAC CCA AAG A

*MMP9*	Forward	GGG ACG CAG ACA TCG TCA TC
	Reverse	TCG TCA TCG TCG AAA TGG GC

## Data Availability

The data used to support the findings of this study are available from the corresponding author upon request.
